# Dietary intake and household exposures as predictors of urinary concentrations of high molecular weight phthalates and bisphenol A in a cohort of adolescents

**DOI:** 10.1038/s41370-021-00305-9

**Published:** 2021-02-22

**Authors:** Anna R. Smith, Katherine R. Kogut, Kimberly Parra, Asa Bradman, Nina Holland, Kim G. Harley

**Affiliations:** 1 Division of Environmental Health Sciences, School of Public Health, University of California, Berkeley, Berkeley, CA USA; 2 Center for Environmental Research and Children’s Health (CERCH), School of Public Health, University of California, Berkeley, Berkeley, CA USA; 3 Department of Epidemiology and Biostatistics, Mel & Enid Zuckerman College of Public Health, University of Arizona, Tucson, AZ USA; 4 Department of Public Health, School of Social Sciences, Humanities and Arts, University of California, Merced, CA USA

**Keywords:** Dietary exposures, Benzylbutyl phthalate, Di-2-ethylhexyl phthalate, Dioctyl phthalate, Bisphenol-A, Endocrine disruptors

## Abstract

**Background:**

Phthalates and bisphenol A (BPA) are endocrine disrupting chemicals used in consumer products, building materials, and food processing and packaging materials. They are associated with adverse health outcomes, especially when exposure occurs during heightened windows of susceptibility.

**Objective:**

We evaluated the relationship between housing and dietary characteristics and the concentration of several high-molecular-weight (HMW) phthalate metabolites and BPA in a cohort of Latina adolescents.

**Methods:**

We collected information on recent food consumption and housing characteristics and quantified the concentration of HMW phthalate and BPA metabolites in urine collected at two different time points. We used generalized estimating equations (GEE) to assess predictors of each metabolite.

**Results:**

No significant associations were observed between housing and dietary characteristics and metabolites of di(2-ethylhexyl) phthalate (DEHP) or BPA. In contrast, higher urinary monobenzyl phthalate (MBzP) concentration was associated with living in a home with vinyl or linoleum flooring (66.7% change, *p*-value <0.01), while higher urinary mono(3-carboxypropyl) phthalate (MCPP) concentration was associated with recent consumption of coffee (47.2% change, *p*-value <0.01), and fast food (30.3% change, *p*-value <0.05).

**Significance:**

These findings may be useful in targeting interventions that reduce phthalate uptake in young adults.

## Introduction

Phthalates and bisphenol A (BPA) are endocrine disrupting chemicals (EDCs) [1] associated with numerous adverse health outcomes such as reproduction and developmental dysfunction [2, 3], metabolic disease [4–7], neurobehavioral development [8, 9], and immune dysfunction [10, 11], especially when exposure occurs during critical windows of susceptibility. Phthalates can be classified into two categories based on their molecular weight (high molecular weight (HMW) and low molecular weight (LMW) phthalates) [12]. While LMW phthalates are used in personal care products and cosmetics as fragrance carriers and solvents [13, 14], HMW phthalates are added to polyvinyl chloride (PVC) plastic to increase durability and flexibility in food processing and packaging materials [15, 16], building materials [17], and medical devices [18]. Individuals are primarily exposed to HMW phthalates via ingestion of food contaminated through processing or packaging [19], as well as via inhalation in buildings containing PVC flooring or other materials [20–22].

Di(2-ethylhexyl) phthalate (DEHP) was historically the most commonly used HMW phthalate, making up about 50% of the market [23], although use has declined in recent years. Human biomonitoring data show that exposure to DEHP and butyl benzyl phthalate (BBzP) has declined [24], reflecting a manufacturing shift toward substitutes, including alternative HMW phthalates such as dioctyl phthalate (DOP), diisononyl phthalate (DiNP), and di-isodecyl phthalate (DiDP).

BPA is a key component in hard polycarbonate plastics. It is used in epoxy resins for consumer and food product packaging, including canned food [25], and it is also found in plastic dinnerware, dental sealants [26], and thermal receipts [27]. Humans are exposed via oral, inhalation, and dermal exposure, although oral exposure from dietary sources is the most common [28].

Many studies have found an association between living in a home with vinyl flooring and higher urinary concentrations of BBzP metabolites [20, 29–31]. One study found that adolescents were primarily exposed to BBzP from food sources, while infants were more commonly exposed through household dust [32].

Fast food is also associated with HMW phthalate exposure. A study conducted in the 2003–2010 National Health and Nutrition Examination Survey (NHANES) cohort observed a positive, dose–response relationship between fast food intake and exposure to HMW phthalates, but not BPA [33]. In a study of 491 Mexican-American pregnant women from the same geographic location as those in the present study, consumption of hamburgers was measured as a proxy for fast food consumption. Participants who consumed hamburgers three or more times a week had 20% higher urinary BPA concentration than non-consumers [34]. Food monitoring and diet studies conducted in Japan found that use of disposable PVC gloves during the preparation and packaging of meals was a major source of dietary intake of DEHP and that sterilizing the gloves with alcohol increased its migration [35].

HMW phthalate exposure is also linked to consuming food from other sources away from home, such as full service restaurants and cafeterias. A study conducted in Italy compared concentrations of DEHP and DiNP in school meals before and after the food was packaged and found that packaging increased phthalate concentrations by over 100% [36]. A recent cross-sectional study of NHANES participants between 2005 and 2014 found that dining out was positively associated with potency-adjusted exposure to multiple phthalates across age groups in the U.S. general population. Among adolescents, consuming food away from the home the prior day was associated with as much as 55% higher cumulative phthalate exposure compared to eating food at home [37]. For the first time, the study showed that the contribution of certain food sources varies by age group. Cafeteria food was associated with a smaller percent difference in phthalate exposure among children, compared to adolescents and adults, despite children consuming two and seven times the amount of cafeteria food as adolescents and adults, respectively [37].

Although several studies have evaluated predictors of phthalate and phenol exposure during pregnancy and childhood, fewer have examined predictors in adolescents. We aimed to assess dietary and household characteristics relevant to HMW phthalate and BPA exposure in a well-characterized cohort of Mexican-American teenage girls in Salinas, CA. We hypothesized that recent consumption of heavily processed and packaged foods, as well as the presence of PVC products in the home, would be associated with higher concentrations of HMW phthalate metabolites. We also hypothesized that recent exposure to canned food and beverages, fast food, and receipts would be associated with higher urinary concentrations of BPA.

## Methods

### Study population

The Health and Environmental Research on Makeup of Salinas Adolescents (HERMOSA) Study was a youth-led intervention study to reduce LMW phthalate and phenol exposure from cosmetics among adolescent girls [13, 14, 38]. Although HMW phthalates and BPA were measured, the intervention did not address these chemicals. Briefly, study participants were 100 Latina adolescent girls living in Salinas, California, recruited through word of mouth and personal networks. Participants were eligible for the study if they were between 14 and 18 years old, spoke English or Spanish, and had lived in the United States for at least 1 year. Data collection took place in June and July 2013. Written informed consent was obtained from parents, and assent was obtained from each teenage participant. All study activities were approved by the Committee for the Protection of Human Subjects at the University of California, Berkeley.

### Data collection

Study participants were interviewed three times: during an initial home visit, where consent/assent was obtained, and at pre- and post-intervention office visits. The pre- and post-intervention visits were exactly 3 days apart during which time participants used only personal care products labeled free from LMW phthalates, parabens, triclosan, and benzophenone-3. The product intervention demonstrated reduced urinary concentrations of these chemicals [14]. The intervention did not require any changes to diet or other household exposures of interest, and urinary concentrations of HMW phthalates and BPA were not significantly different between the pre- and post-intervention visits (see Table S3 from Harley et al. [14]).

During the initial home visit, a participant (typically the parent) completed a usual practice questionnaire related to family and housing characteristics, as well as household cooking and cleaning habits.

Youth participants were provided a 24-h food diary on which they were instructed to list every food and beverage consumed the full day prior to and on the day of their pre-intervention office visit. They were asked to list the ingredients included in meals (e.g., the components of their burrito), to star any food or beverages that came from a can, and to note the origin of each meal consumed (i.e. home prepared, restaurant name, school, etc.). Participants handed in their first 24-h food diary at the pre-intervention office visit and were provided a second food diary to complete in the same way prior to the post-intervention office visit.

During both office visits, participants also completed a recent practice questionnaire asking them about their behaviors on the day of the office visit, including specific questions regarding whether they handled a receipt in the past 24 h or consumed a canned food or drink item that day. The questionnaire asked if participants had consumed specific breakfast items that day (cold cereal, eggs, or breakfast meat), specific lunch items (hamburger, french fries, pizza, burrito or taco, chicken, cold cuts or deli meats, tuna fish, or soup), or specific drink items (milk, 100% juice, sweetened juice, ice tea, soda, coffee or hot tea, or water). Participants were encouraged to look at their food diary form to help remember.

At each office visit, the participant provided a spot urine sample in a sterile polypropylene cup for measurement of phthalate and BPA analytes.

### Dietary and household predictors

Each family’s usual practices were ascertained at the home visit and included whether or not participants usually stored leftovers or microwaved in plastic, whether there was a plastic or vinyl shower curtain in the home, and whether the home had any linoleum and/or vinyl flooring. Although vinyl flooring contains phthalates and linoleum flooring does not [39], preliminary research showed that participants were unlikely to know whether their floors were vinyl or linoleum, and so this was asked as a single category. Whether or not each participant operated a cash register at work was ascertained at the office visits from the questionnaire.

Recent practices (past 1–12 h) (“Foods Today,” “Drinks Today,” “Canned Items Today”, and “Recent Receipt Contact”) were ascertained from the recent practice questionnaires and chosen a priori based on the literature. “Recent receipt contact” (past 24 h) was also ascertained from recent practice questionnaire. The food and drink items were limited to those in which a sufficient frequency of the population had consumed that day (i.e. *N* > 5 for both a “yes” and “no” response). If participants had responded “yes” to eating breakfast that day, they were then asked if they had consumed specific breakfast, lunch, drink, and canned items listed in the prior section. The office visits took place early morning to mid-afternoon, and so some participants had only consumed breakfast, while others had consumed both breakfast and lunch the day of the visit. Dietary intake (past 1–12 h) from the questionnaires was used for most analyses, because it was more difficult to ascertain food categories from the food diary data, although dietary intake recorded on the food diary was examined in supplemental analyses.

The origin of foods (past 24 h) (i.e. fast food, school meal) was obtained from the participants’ 24-h food diaries. Participants were asked to provide the location of each meal, which included the specific restaurant name, if applicable. Based on their responses, “fast food” and “school meal” binary variables were created, which indicate whether a participant had consumed any fast food or school meal, respectively, in the past 24 h. At present, there is no agreed upon definition of “fast food” for research purposes [40]. The NHANES definition was used in the present study, which classifies fast food as: (1) food obtained from restaurants without waiter/waitress service, or (2) food from pizza restaurants, regardless of waiter/waitress service, or (3) carry out and delivery food [33].

### Laboratory analysis

Urine specific gravity was measured in the field at the time of collection using a handheld refractometer (PAL-10S, Atago USA Inc.). All urine samples were then aliquoted and frozen at −80 °C until shipment on dry ice to the Environmental Health Laboratory of the California Department of Public Health in Richmond, California, for analysis.

Six urinary HMW phthalate metabolites were measured and included in the analysis: monobenzyl phthalate (MBzP), a metabolite of BBzP; mono-2-ethylhexyl phthalate (MEHP), mono-(2-ethyl-5-hydroxyhexyl) phthalate (MEHHP), mono-(2-ethyl-5-oxohexyl) phthalate (MEOHP), and mono-(2-ethyl-5-carboxypentyl) phthalate (MECCP), all metabolites of DEHP; and mono(3-carboxypropyl) phthalate (MCPP), a metabolite of DOP and a non-specific metabolite of several HMW phthalates.

Urinary phthalate metabolites were quantified using a high-performance liquid chromatography/tandem mass spectrometer (HPLC-MS/MS) (API 5000; AB Sciex). As described previously [14], a mixture of stable isotope-labeled internal standards (Cambridge Isotopes) was added to samples. The spiked samples were digested with glucuronidase at 37 °C for 90 min. After digestion, the samples were injected into an on-line solid-phase extraction column. The analytes were separated chromatographically on a Betasil™ phenyl column in a mobile phase, which consisted of acetonitrile and 0.1% acetic acid, in gradient elution mode [41]. MS/MS data were acquired using multi-period mode during the chromatographic elution time. An electrospray ionization source, operating in negative mode, ionized the analytes.

Urinary BPA concentrations were quantified using a reverse-phase HPLC-MS/MS system (API 5500 QTRAP; AB Sciex) as described previously [42]. Urine samples were spiked with stable isotope-labeled internal standards. The spiked samples were enzymatically de-conjugated overnight at 37 °C. After digestion, the samples were processed by solid-phase extraction using C18 cartridges. The eluents were evaporated and reconstituted with mobile phase prior to analyses. Atmospheric pressure chemical ionization ionized analytes.

The *r*^2^ of each of the analyte calibration curves was ≥0.99. Randomly selected samples were analyzed in duplicate. The relative percent difference between duplicates ranged from 0 to 19.6% for samples greater than the limit of detection (LOD). Quality control samples were included in every analytical run. Recoveries were within 30% of the respective target values. All analytes had a coefficient of variation (CV) of ≤15%, indicating that the precision for each quality control level was sufficient. For field quality control, 20 blanks were collected using highly purified water in contact with all field collection materials. Analytes in the field blanks were below their respective LODs.

Concentrations below the LOD were assigned the value of LOD/(square root 2) [43]. Chemical analyte concentrations were reported in ng/mL of urine. To adjust for variability in urine output, dependent on the hydration state of the participant at the time of sample collection, urinary concentrations were corrected for specific gravity, using the equation: (analyte concentration × [1.018–1]) / (sample specific gravity–1), where 1.018 is the median specific gravity for the population [44]. The HMW phthalate metabolites and BPA were log-normally distributed and log_2_-transformed for analysis.

### Statistical analysis

The main outcomes of interest were urinary concentrations of BPA, MBzP, MCPP, and the DEHP metabolites. Because DEHP metabolizes to four different metabolites, a single variable for the molar sum of the DEHP metabolites (ΣDEHP) was created by dividing each metabolite concentration by its molecular weight and then summing: [(MEHP × (1/278.348)) + (MEHHP × (1/294.347)) + (MEOHP × (1/292.331)) + (MECPP × (1/308.33))]. Uncorrected and specific gravity-corrected geometric means (GM), distributions, and detection frequencies were calculated for each of the urinary metabolites of HMW phthalates and BPA. Because all metabolite concentrations were log-normally distributed, we log-transformed these variables using a log_2_ transformation, which was more appropriate to the range of the concentrations than a log_10_ or natural log. To examine how GM concentrations in this cohort compare to those of a nationally representative sample, urinary HMW phthalate and BPA concentrations were compared to unweighted concentrations for female 12–19 year olds participating in the 2013–2014 wave of the NHANES (*N* = 235) using two-sample *t*-tests of log_2_-transformed concentrations [45, 46].

Dietary and household predictors of urinary MBzP, ΣDEHP, MCPP, and BPA concentrations were examined in the HERMOSA dataset using generalized estimating equations (GEE) models to account for repeated measures on each individual. Each model was mutually adjusted for all predictors, as well as for age, body mass index (BMI), and time since last meal. It was assumed that time since last meal would influence the magnitude of urinary concentration of each of the metabolites, due to their short half-lives [47]. A sample size of 200 was used (two office visits each for 100 participants), and the GEE models accounted for non-independence of the two visits within the same participant. An exchangeable correlation structure was chosen, assuming correlations were constant between any two observations on the same subject. A single set of a priori predictors was used for all HMW phthalate metabolite models, while a different set of a priori predictors was used for the BPA model. Percent change in concentration of urinary metabolites was calculated for each predictor from the regression coefficient (expressed as log_2_), using the equation (2^Coefficient^−1) × 100. In addition, we calculated the GM of phthalate and BPA metabolite concentrations among those with and without significant predictors. Finally, we ran sensitivity analyses in which we ran equivalent GEE models using food diary data, instead of questionnaire data, to ascertain participant’s recent practices (past 1–12 h).

## Results

All participants were Latina girls between the ages of 14 and 18 years old. The majority of participants in the study were born in the United States (81%), lived in Spanish-speaking households (86%), and were low income (67% were from households with annual incomes less than or equal to $36,000). The majority of the participants’ parents did not graduate from high school. Most participants had a normal BMI (56%) (Table 1). All the participants were interviewed in English, while their parents were interviewed in either English or Spanish.

**Table 1:** Demographic characteristics of HERMOSA Study participants (*N* = 100).

Characteristic	*N*	(%)
Age (years)		
14	11	(11%)
15	22	(22%)
16	29	(29%)
17	30	(30%)
18	8	(8%)
Country of birth		
United States	81	(81%)
Mexico	19	(19%)
Language spoken at home		
Mostly English	14	(14%)
Mostly Spanish	57	(57%)
Both Spanish and English	29	(29%)
Annual household income		
≤$24,000	38	(38%)
$24,000-$36,000	29	(29%)
>$36,000	25	(25%)
Unknown/refused	8	(8%)
Parental highest education		
6th grade or less	25	(25%)
7–12th grade	32	(32%)
High school graduate	33	(33%)
Unknown	10	(10%)
BMI		
Underweight	4	(4%)
Normal weight	56	(56%)
Overweight	26	(26%)
Obese	14	(14%)

The majority of participants reported using plastic containers for leftovers (94%). Half of the participants reported microwaving leftovers in plastic containers (50%) and living in a home with linoleum or vinyl flooring (50%). The majority of participants reported living in a home with a plastic or vinyl shower curtain (75%), while few reported operating a cash register (a marker for handling a large volume of receipts) at work (7%) (Table 2). A small percentage of individuals reported consumption of breakfast meat, eggs, chicken, and cold cuts the day of each office visit (<10%). The majority of participants drank bottled water (60%), and almost half of the participants consumed milk (48%) the day of each office visit (Table 2). In addition, about one-third of the participants consumed fast food in the 24 h leading up to the office visit (38%) and/or touched a receipt (42%) (Table 2).

**Table 2:** Frequency of reported dietary and household exposures for HERMOSA study participants for usual practices (asked at home visit) and recent practices (asked at office visits 1 and 2).

Exposure	Percentage of study population
Family’s Usual Practices	
Plastic containers for leftovers	94%
Microwave in plastic	50%
Linoleum or vinyl floors in home	50%
Plastic or vinyl shower curtain	75%
Operate cash register at work	7%
Participant’s Recent Practices (past 1–12 h)^a^	
Foods Today (yes/no)	
Breakfast meat	7%
Eggs	9%
Chicken (including sandwich)	8%
Cold cuts or deli meats	5%
Drinks Today (yes/no)	
Coffee or hot tea	8%
100% fruit juice	15%
Sweetened fruit drink	8%
Milk	48%
Bottled water	60%
Canned Items Today (yes/no)	
Any canned food or drink	9.5%
Participant’s Recent Practices (past 24 h)^a^	
Receipt Contact	
Receipt today or yesterday	42%
Meal Location	
Fast food today or yesterday	38%
School meal today or yesterday	12%

^a^*N* = 200 because asked at office visits 1 and 2.

All HMW phthalate urinary metabolites were detected in over 90% of the participant samples, while BPA was detected in over 80% of participant samples. The distributions of urinary metabolite concentrations are summarized in Table 3. Mean log_2_-transformed urinary phthalate concentrations in the study population were either similar or lower than those of the nationally representative sample of female 12–19 year olds participating in the 2013–2014 wave of the NHANES. There was no significant difference between MBzP and MEHP metabolites in the two study populations. In contrast, MEHHP, MECPP, MEOHP, MCPP, and BPA metabolites were significantly lower in the HERMOSA study, compared to the female 12–19-year-old NHANES participants.

**Table 3:** Distribution of uncorrected (ng/mL) and specific gravity corrected (italics) urinary analyte concentration in HERMOSA study participants.

Percentiles						
Metabolite	>LOD (%)	GM	25%	50%	75%	95%
MBzP^a^	99.5	4.7	2.3	5.6	10.4	32.1
		*6.6*	*3.8*	*6.4*	*10.6*	*31.3*
MEHP^b^	90.5	1.4	0.6	1.5	3.4	8.2
		*2.0*	*1.0*	*2.1*	*3.4*	*9.0*
MEHHP^b^	98.5	4.5	2.3	5.2	9.3	21.9
		*6.3*	*3.9*	*6.0*	*9.6*	*22.8*
MECPP^b^	99.5	8.6	4.9	9.3	18.7	36.3
		*12.0*	*7.6*	*11.9*	*17.7*	*40.1*
MEOHP^b^	99.0	3.6	1.9	4.0	7.7	15.9
		*5.1*	*3.2*	*4.8*	*7.6*	*16.0*
∑DEHP	-----	----	----	----	----	----
		*25.5*	*15.9*	*24.2*	*37.8*	*87.7*
MCPP^c^	98.0	1.3	0.7	1.6	2.7	6.7
		*1.9*	*1.2*	*1.6*	*2.6*	*7.0*
BPA	81.5	0.8	0.3	0.9	1.9	5.9
		*1.1*	*0.7*	*1.2*	*2.0*	*5.6*

^a^Metabolite of BzBP.

^b^Metabolite of DEHP.

^c^Metabolite of DOP, DiNP, DiDP.

Higher urinary MBzP concentrations were seen among adolescent girls living in a home with vinyl or linoleum flooring (66.7% change, *p*-value <0.01), or who usually used plastic containers for leftovers (71.4% change, *p*-value <0.10) (Table 4). Higher urinary MCPP concentrations were associated with several recent exposures, which included consuming chicken (58.5% change, *p*-value <0.10), consuming cold cuts (87.9% change, *p*-value <0.10), drinking coffee (47.2% change, *p*-value <0.01), and eating fast food (30.3% change, *p*-value <0.05). There were no strong associations between urinary DEHP metabolite concentration and usual or recent exposures.

**Table 4:** GEE population-averaged model for predictors of urinary concentration of phthalate metabolites (ng/mL) in HERMOSA participants.

Phthalate metabolites						
	MBzP		∑DEHP		MCPP	
Predictor	% Change^a^	(95% C.I.)	% Change^a^	(95% C.I.)	% Change^a^	(95% C.I.)
Family’s Usual Practices						
Plastic containers for leftovers	71.4*	(4.6, 138.2)	8.3	(−21.1, 37.6)	−18.1	(−65.0, 28.7)
Microwave in plastic	25.6	(−3.6, 54.7)	0.7	(−26.5, 28.0)	1.2	(−22.5, 24.8)
Linoleum or vinyl floors in home	66.7***	(35.9, 97.5)	1.5	(−25.9, 28.8)	15.1	(−8.4, 38.6)
Plastic or vinyl shower curtain	10.9	(−25.4, 47.2)	−12.2	(−44.5, 20.1)	−18.1	(−44.9, 8.7)
Participant’s Recent Practices (past 1–12 h)						
Foods Today						
Breakfast meat	−13.8	(−57.3, 29.7)	17.3	(−49.6, 84.3)	−2.7	(−49.0, 54.4)
Eggs	15.1	(−28.8, 59.1)	−1.3	(−47.0, 44.3)	−10.7	(−57.5, 36.1)
Chicken	4.5	(−34.1, 43.2)	4.7	(−45.3, 54.7)	58.5*	(5.1, 111.9)
Cold cuts or deli meats	−16.2	(−72.6, 40.2)	11.5	(−43.0, 66.1)	87.9*	(3.7, 172.0)
Drinks Today						
Coffee or hot tea	−13.9	(−54.9, 27.2)	4.9	(−35.1, 45.0)	47.2***	(21.7, 72.8)
100% fruit juice	−14.4	(−37.5, 8.7)	−0.3	(−34.9, 34.4)	20.8	(−9.6, 51.2)
Sweetened fruit drink	15.3	(−22.4, 53.0)	12.7	(−31.1, 56.4)	−9.3	(−51.2, 32.5)
Milk	−2.6	(−28.5, 23.3)	11.3	(−14.4, 37.0)	17.5	(−6.5, 41.5)
Bottled water	−5.8	(−26.1, 14.5)	−10.8	(−33.3, 11.6)	−2.9	(−26.0, 20.3)
Participant’s Recent Practices (past 24 h)						
Meal Location						
Fast food today or yesterday	−9.0	(−27.4, 9.5)	14.2	(−5.3, 33.8)	30.3**	(7.0, 53.5)
School meal today or yesterday	−7.4	(−41.2, 26.3)	−17.1	(−59.0, 24.9)	23.8	(−13.5, 61.0)

*p-Value < 0.10; **p-value < 0.05; ***p-value < 0.01.

^a^Model mutually adjusted for all predictors in table, plus age, BMI, and time since last meal.

The absolute change, rather than the percent change, associated with these predictors is shown in Supplemental Table 1, which presents the GM of HMW phthalate urinary analyte concentrations (ng/mL) by (yes/no) exposure of predictors with *p*-value <0.10 in GEE models. Urinary MBzP concentration GM among participants living in a home with vinyl or linoleum flooring was 8.7 ng/mL, compared to 5.0 ng/mL among participants who did not. The GM among participants who usually used plastic containers for leftovers was 6.8 ng/mL, compared to 3.9 ng/mL among participants who did not. Urinary MCPP concentration GM among participants who had recently consumed chicken, cold cuts, and coffee was 3.1 ng/mL, 3.9 ng/mL, and 2.4 ng/mL, respectively, compared to 1.8 ng/mL among those who did not. The GM among participants who had recently consumed fast food was 2.2 ng/mL, compared to GM 1.7 ng/mL among those who had not.

Urinary BPA concentrations were associated with operating a cash register at work (65.2% change, *p*-value <0.10). In contrast, there was no association between any recent practice predictor and BPA concentration (Table 5). GM BPA urinary analyte concentrations (ng/mL) by (yes/no) exposure of predictors with *p*-value <0.10 in GEE models are reported in Supplemental Table 1. Urinary BPA GM among participants who operate a cash register at work was 1.7 ng/mL, compared to 1.1 ng/mL among those who did not.

**Table 5:** GEE population-averaged model for predictors of urinary concentration of BPA (ng/mL) in HERMOSA participants.

Predictor	% Change^a^	(95% C.I.)
Family’s Usual Practices		
Plastic containers for leftovers	33.9	(−26.8, 94.6)
Microwave in plastic	6.6	(−32.4, 45.8)
Operate cash register at work	65.2*	(1.1, 129.3)
Participant’s Recent Practices (past 1–12 h)		
Foods Today		
Breakfast meat	−38.6	(−132.8, 55.7)
Eggs	15.4	(−47.4, 78.2)
Chicken (including sandwich)	−21.2	(−86.0, 43.5)
Cold cuts or deli meats	−13.6	(−130.3, 103.1)
Drinks Today		
Coffee or hot tea	−25.9	(−91.3, 39.5)
100% fruit juice	12.2	(−33.2, 57.6)
Sweetened fruit drink	−18.6	(−98.7, 61.5)
Milk	−6.4	(−44.7, 31.9)
Bottled water	−2.1	(−37.0, 32.8)
Canned Items Today (yes/no)		
Any canned food or drink	43.2	(−6.1, 92.5)
Participant’s Recent Practices (past 24 h)		
Receipt Contact		
Touched receipt today or yesterday	−10.4	(−48.6, 27.9)
Meal Location		
Fast food today or yesterday	23.1	(−10.6, 56.9)
School meal today or yesterday	−21.8	(−79.2, 35.7)

*p-Value < 0.10.

^a^Model mutually adjusted for all predictors, plus age, BMI, and time since last meal.

In sensitivity analyses, in which we used food diary data to ascertain participants’ recent dietary practices (past 1–12 h), instead of the questionnaire given on the day of each office visit, similar results were observed for MBzP and DEHP metabolites, but coffee consumption was no longer significantly associated with MCPP metabolite.

## Discussion

We found that higher MBzP urinary concentration was associated with vinyl/linoleum flooring in the home, while higher MCPP concentration was associated with recent dietary consumption of coffee and/or hot tea and fast food.

Prior studies have demonstrated adverse health effects associated with exposure to phthalates and BPA during adolescence. For example, a recent cross-sectional study of 205 adolescents in the New Bedford Cohort found an association between an increase in urinary concentration of the molar sum of 11 phthalate metabolites, derived from antiandrogenic parent compounds, and attention-deficit/hyperactivity disorder (ADHD)-related behavior problems [48]. Another cross-sectional study in a subset of 2003–2004 NHANES participants (8–15 years) found that higher urinary BPA concentration was associated with ADHD [49]. A cross-sectional study conducted in Chinese school children (8–15 years) found that nine urinary phthalate metabolites and five molar sums were positively associated with BMI and/or waist circumference [50]. Urinary BPA concentration was also associated with obesity in children and adolescents in a cross-sectional study of 2003–2008 NHANES participants (6–19 years) [51]. In addition, a study conducted in adolescents in Belgium (14–15 years) found a positive association between MnBP (metabolite of DBP) and ΣDEHP and asthma diagnosis, as well as 8-OHdG, a biomarker of oxidative stress [52].

The association between higher urinary concentrations of MBzP and living in a home with vinyl/linoleum flooring in our study is consistent with prior studies [20, 29, 30, 53]. For instance, one study found that living in a home with PVC flooring in the kitchen and master bedroom, compared to no PVC, was associated with increased concentration of MBzP (99.9%, *p*-value <0.0001) in the first trimester urine of pregnant women [20]. The creatinine-corrected GM of MBzP in the study was similar to that of the specific-gravity corrected GM of MBzP in the HERMOSA study. Another study in Swedish mothers and children (ages 6–11 years old) found that living in a house with PVC in floorings or wall coverings was associated with higher levels of MBzP (*β* = 0.48, 95% CI: 0.04, 0.91). The GM levels (μg/g creatinine) for those with PVC floorings, compared to those without, were 17.47 and 7.49 [30].

Finally, a prospective cohort study in New York City of 239 children reported that “vinyl or linoleum” flooring in the home environment was observed in 135 (56%) of monitored rooms, and that these rooms had significantly higher indoor air GM concentrations of BBzP (23.9 ng/m^3^) than rooms with wood or carpet flooring (10.6 ng/m^3^). Children from homes with “vinyl or linoleum” flooring had significantly higher urinary BBzP metabolite concentrations (GM: 32.6; interquartile range (IQR): 15.5, 70.9 ng/mL), compared to children with carpet or wood flooring (GM: 18.3, IQR: 9.1, 37.2 ng/mL), while indoor air DEHP was not associated with flooring type [53].

In our study, participants were asked whether they had linoleum or vinyl flooring as a proxy for vinyl flooring. This likely resulted in exposure misclassification, as phthalates would be expected in vinyl and not linoleum flooring products. However, this exposure misclassification would have likely biased estimates toward the null. Regardless, we still found a significant association between this predictor and MBzP concentration.

We found that consuming fast food the day of or day prior to the office visit was associated with higher MCPP concentrations. Fast food consumption at least once per week, compared to less than once per week, was associated with higher urinary concentrations of metabolites of BBzP (55%, 95% CI: 9, 123) and DiNP (35%, 95% CI: −2, 85) in a cohort of U.S. children in Ohio [54]. A study examining the 2003–2010 NHANES cohort found that both fast food intake and fast food-derived fat intake was positively associated with metabolites of DEHP and DiNP [33]. Although our study was not able to measure specific metabolites of DiNP, this compound likely also metabolizes to MCPP, which was associated with fast food consumption in this study.

In an exploratory analysis of data collected as part of the 2003–2004 NHANES, total dairy consumption (assessed from a 24-h dietary recall) was significantly associated with higher log-transformed creatinine-adjusted MCPP concentrations (*β* = 0.039, 95% CI: 0.021, 0.057) [55]. In our analysis, higher urinary MCPP concentration was associated with coffee and/or hot tea the day of the office visit, which does sometimes contain milk, but also could be due to packaging or processing. The consumption of milk alone was not a strong predictor of MCPP concentration in our models. In the same NHANES study, poultry consumption was a significant predictor of individual and ΣDEHP metabolites (*β* = 0.048, 95% CI: 0.021, 0.075) [55]. In our analysis, only MCPP urinary concentration was associated with poultry consumption. The differences between analyses could be due to changes in HMW phthalate use [24], as the NHANES study was conducted about a decade ago. A cross-sectional study examined the relationship between food and caloric intake, assessed by 24-h dietary recalls, and urinary phthalate metabolites in 2743 children and adolescents (ages 6–19) from the NHANES 2003–2008 cohort and observed an association between higher consumption of meat, poultry, and fish and increased HMW metabolite (% increment/unit consumption 0.09, 95% CI: 0.02, 0.16) and ΣDEHP metabolite (0.09, 95% CI: 0.02, 0.17) [56]. Animal products are a potential source of phthalates from processing and packaging [15, 56–58]. Consistently, analyses of milk in Belgium found higher concentrations of DEHP and BBzP in milk retail products than in raw cow milk [59].

Unlike other studies, we did not find that specific food consumption was associated with higher urinary concentrations of DEHP metabolites. Many studies have examined dietary predictors of DEHP metabolites, because DEHP was historically the most common phthalate plasticizer and prevalent in food products. However, marketplace trends in phthalate use are changing as a result of recent legislation. DEHP is increasingly being replaced with DiNP and DiDP in products. As of 2012, these two phthalates combined account for 30–60% of the current plasticizer market in the United States and the European Union [24]. These market trends are reflected in U.S. biomonitoring data showing that ΣDEHP metabolites decreased by 37% and MCOP (a DiNP metabolite) increased by 149% between 2001 and 2010 [24]. Thus, the lack of strong associations between ΣDEHP urinary metabolite concentration and dietary predictors in our study may reflect changing trends.

BPA is used in polycarbonate plastics and epoxy resins, which are present in the inner coatings of cans and can migrate from cans and other plastics into food [60]. BPA is also used in certain cash register and point-of-contact receipts [61]. We hypothesized recent canned food and beverage consumption, as well as recent receipt contact, would be associated with increased urinary BPA concentration. There was no association between recent receipt contact or recent canned food and beverage consumption. In contrast, there was a slight association between operating a cash register and higher BPA concentration in our study. In a Swedish cohort of mothers and children in 2006–2008, there was no association between urinary BPA concentration and canned food or beverage consumption [62], although another study from a 2003–2006 cohort found a positive association between urinary BPA concentration and canned vegetable consumption frequency in U.S. men and women [63]. A study characterizing dietary BPA exposure in the French population in 2005 found that canned products accounted for about 50% of total BPA exposure [64]. Differences could be due to some companies phasing out BPA with other understudied chemicals such as bisphenol S and bisphenol F [65].

Findings from a study examining whether increased contact with thermal paper receipts is associated with increased BPA excretion in NHANES participants (2003–2004) [66] was consistent with the present study findings. In the NHANES study, females with potential occupational exposure to thermal paper receipts had higher levels of urinary BPA (GM: 5.45, 95% CI: 4.02, 7.39 μg/L) compared to females with unlikely occupational exposure (GM: 2.16, 95% CI: 1.73, 2.70 μg/L) [66]. In the present study, females who operate a cash register at work had higher urinary BPA concentration than those who do not (GM: 1.7 ng/mL, compared to GM: 1.1 ng/mL). However, receipt contact in the past 24 h was not associated with BPA concentration, suggesting that there could be additional occupational BPA exposures in cash register jobs, or that the duration and magnitude of receipt contact may have an impact (i.e. persistent handling of receipts versus touching a receipt).

One limitation of the present study is that we were not able to measure more metabolites of substitution chemicals, such as DiNP, DiDP, DINCH, BPS, and BPF. In addition, there were not enough participants eating certain food items of interest, such as pizza and hamburgers, to proceed with these analyses. The dietary items selected for the models were chosen based on prior knowledge and whether enough participants consumed them.

The major strength of our study was that it examined predictors of urinary concentration of MCPP, a metabolite of DOP and other HMW phthalates, which are replacing DEHP and are relatively understudied in the literature. The study was also specifically designed to obtain detailed, individual level data on sources and timing of dietary and household exposures within the past 24 h to examine associations with phthalate and phenol concentrations in adolescents.

This is the first study to examine sources of HMW phthalate and BPA in adolescents, specifically in a Mexican-American population, who may have distinct dietary and behavioral patterns. By examining the relationship between urinary concentrations of HMW phthalates and BPA metabolites and specific dietary and household exposure predictors in population subsets, the most significant exposure sources can be pinpointed in order to guide product regulation and consumer recommendations. This is important in providing guidance for pregnant women, children, and adolescents, because exposure to EDCs during these critical windows can interfere with normal developmental processes and result in adverse health effects later in life.

While urinary MBzP concentration was associated with household factors, MCPP concentration was associated with recent dietary exposures. There were no strong associations between dietary and household predictors and DEHP metabolites and BPA. Adolescence is a critical period of development and growth, during which sensitivity to the adverse effects of EDCs increases [67]. Interventions that remove PVC gloves, tubing, and containers from food manufacturing and production processes, as well as replace vinyl and linoleum flooring in homes, could be beneficial in reducing exposure to HMW phthalates.

## Supplementary information

Supplemental Table 1

## References

[1] Latini G (2005). Monitoring phthalate exposure in humans. Clin Chim Acta.

[2] Berger K, Eskenazi B, Kogut K, Parra K, Lustig RH, Greenspan LC, et al. Association of prenatal urinary concentrations of phthalates and bisphenol A and pubertal timing in boys and girls. Environ Health Perspect. 2018;126:097004. 10.1289/EHP3424 PMC6375461 30203993

[3] Watkins DJ, Sánchez BN, Téllez-Rojo MM, Lee JM, Mercado-García A, Blank-Goldenberg C (2017). Phthalate and bisphenol A exposure during in utero windows of susceptibility in relation to reproductive hormones and pubertal development in girls. Environ Res.

[4] Tran V, Tindula G, Huen K, Bradman A, Harley K, Kogut K (2017). Prenatal phthalate exposure and 8-isoprostane among Mexican-American children with high prevalence of obesity. J Dev Orig Health Dis.

[5] Harley KG, Berger K, Rauch S, Kogut K, Henn BC, Calafat AM (2017). Association of prenatal urinary phthalate metabolite concentrations and childhood BMI and obesity. Pediatr Res.

[6] Hoepner LA, Whyatt RM, Widen EM, Hassoun A, Oberfield SE, Mueller NT (2016). Bisphenol A and adiposity in an inner-city birth cohort. Environ Health Perspect.

[7] Heggeseth BC, Holland N, Eskenazi B, Kogut K, Harley KG (2019). Heterogeneity in childhood body mass trajectories in relation to prenatal phthalate exposure. Environ Res.

[8] Hyland C, Mora AM, Kogut K, Calafat AM, Harley K, Deardorff J, et al. Prenatal exposure to phthalates and neurodevelopment in the CHAMACOS cohort. Environ Health Perspect. 2019;127:107010. 10.1289/EHP5165 PMC6867166 31652105

[9] Balalian AA, Whyatt RM, Liu X, Insel BJ, Rauh VA, Herbstman J (2019). Prenatal and childhood exposure to phthalates and motor skills at age 11 years. Environ Res.

[10] Zhou A, Chang H, Huo W, Zhang B, Hu J, Xia W (2017). Prenatal exposure to bisphenol A and risk of allergic diseases in early life. Pediatr Res.

[11] Berger K, Eskenazi B, Balmes J, Kogut K, Holland N, Calafat AM (2019). Prenatal high molecular weight phthalates and bisphenol A, and childhood respiratory and allergic outcomes. Pediatr Allergy Immunol.

[12] National Research Council (US) Committee on the Health Risks of Phthalates. Phthalates and cumulative risk assessment: the tasks ahead. US: National Academies Press; 2008. 25009926

[13] Berger KP, Kogut KR, Madrigal DS, Cardenas M, Vera IA, Meza-Alfaro G (2018). Personal care product use as a predictor of urinary concentrations of certain phthalates, parabens, and phenols in the HERMOSA study. J Expo Sci Environ Epidemiol.

[14] Harley KG, Kogut K, Madrigal DS, Cardenas M, Vera IA, Meza-Alfaro G (2016). Reducing phthalate, paraben, and phenol exposure from personal care products in adolescent girls: findings from the HERMOSA Intervention Study. Environ Health Perspect.

[15] Alp AC, Yerlikaya P (2020). Phthalate ester migration into food: effect of packaging material and time. Eur Food Res Technol.

[16] Carlos KS, de Jager LS, Begley TH (2018). Investigation of the primary plasticisers present in polyvinyl chloride (PVC) products currently authorised as food contact materials. Food Addit Contam Part A.

[17] Bornehag C-G, Lundgren B, Weschler CJ, Sigsgaard T, Hagerhed-Engman L, Sundell J (2005). Phthalates in indoor dust and their association with building characteristics. Environ Health Perspect.

[18] Calafat AM, Needham LL, Silva MJ, Lambert G (2004). Exposure to di-(2-ethylhexyl) phthalate among premature neonates in a neonatal intensive care unit. Pediatrics.

[19] Serrano SE, Braun J, Trasande L, Dills R, Sathyanarayana S. Phthalates and diet: a review of the food monitoring and epidemiology data. Environ Health. 2014;13:43. 10.1186/1476-069X-13-43 PMC4050989 24894065

[20] Shu H, Jönsson BAG, Gennings C, Lindh CH, Nånberg E, Bornehag C-G. PVC flooring at home and uptake of phthalates in pregnant women. Indoor Air. 2018;29. https://onlinelibrary.wiley.com/doi/abs/10.1111/ina.12508. 10.1111/ina.12508 30240038

[21] Gaspar FW, Castorina R, Maddalena RL, Nishioka MG, McKone TE, Bradman A (2014). Phthalate exposure and risk assessment in california child care facilities. Environ Sci Technol.

[22] Bi C, Liang Y, Xu Y (2015). Fate and transport of phthalates in indoor environments and the influence of temperature: a case study in a test house. Environ Sci Technol.

[23] Shin I-S, Lee M-Y, Cho E-S, Choi E, Son H-Y, Lee K-Y (2014). Effects of maternal exposure to di(2-ethylhexyl)phthalate (DEHP) during pregnancy on susceptibility to neonatal asthma. Toxicol Appl Pharmacol.

[24] Zota AR, Calafat AM, Woodruff TJ (2014). Temporal trends in phthalate exposures: findings from the National Health and Nutrition Examination Survey, 2001–2010. Environ Health Perspect.

[25] Noonan GO, Ackerman LK, Begley TH (2011). Concentration of bisphenol A in highly consumed canned foods on the U.S. market. J Agric Food Chem.

[26] Löfroth M, Ghasemimehr M, Falk A, von Steyern PV. Bisphenol A in dental materials – existence, leakage and biological effects. Heliyon. 2019;5:e01711. https://www.ncbi.nlm.nih.gov/pmc/articles/PMC6538958/. 10.1016/j.heliyon.2019.e01711 PMC6538958 31193754

[27] Pacyga DC, Sathyanarayana S, Strakovsky RS (2019). Dietary predictors of phthalate and bisphenol exposures in pregnant women. Adv Nutr.

[28] Huang R, Liu Z, Yin H, Dang Z, Wu P, Zhu N (2018). Bisphenol A concentrations in human urine, human intakes across six continents, and annual trends of average intakes in adult and child populations worldwide: a thorough literature review. Sci Total Environ.

[29] Carlstedt F, Jönsson BAG, Bornehag C-G (2013). PVC flooring is related to human uptake of phthalates in infants: phthalate uptake in infants. Indoor Air.

[30] Larsson K, Ljung Björklund K, Palm B, Wennberg M, Kaj L, Lindh CH (2014). Exposure determinants of phthalates, parabens, bisphenol A and triclosan in Swedish mothers and their children. Environ Int.

[31] Hammel SC, Levasseur JL, Hoffman K, Phillips AL, Lorenzo AM, Calafat AM (2019). Children’s exposure to phthalates and non-phthalate plasticizers in the home: The TESIE study. Environ Int.

[32] Wormuth M, Scheringer M, Vollenweider M, Hungerbühler K (2006). What are the sources of exposure to eight frequently used phthalic acid esters in Europeans?. Risk Anal.

[33] Zota AR, Phillips CA, Mitro SD (2016). Recent fast food consumption and bisphenol A and phthalates exposures among the U.S. population in NHANES, 2003–2010. Environ Health Perspect.

[34] Quirós-Alcalá L, Eskenazi B, Bradman A, Ye X, Calafat AM, Harley K (2013). Determinants of urinary bisphenol A concentrations in Mexican/Mexican-American pregnant women. Environ Int.

[35] Tsumura Y, Ishimitsu S, Kaihara A, Yoshii K, Nakamura Y, Tonogai Y (2001). Di(2-ethylhexyl) phthalate contamination of retail packed lunches caused by PVC gloves used in the preparation of foods. Food Addit Contam.

[36] Cirillo T, Fasano E, Castaldi E, Montuori P, Amodio Cocchieri R (2011). Children’s exposure to Di(2-ethylhexyl)phthalate and dibutylphthalate plasticizers from school meals. J Agric Food Chem.

[37] Varshavsky JR, Morello-Frosch R, Woodruff TJ, Zota AR (2018). Dietary sources of cumulative phthalates exposure among the U.S. general population in NHANES 2005–2014. Environ Int.

[38] Madrigal DS, Minkler M, Parra KL, Mundo C, Gonzalez JEC, Jimenez R (2016). Improving Latino youths’ environmental health literacy and leadership skills through participatory research on chemical exposures in cosmetics: The HERMOSA Study. Int Q Community Health Educ.

[39] Kolarik B, Naydenov K, Larsson M, Bornehag C-G, Sundell J (2008). The association between phthalates in dust and allergic diseases among Bulgarian children. Environ Health Perspect.

[40] Chou S-Y, Grossman M, Saffer H (2004). An economic analysis of adult obesity: results from the Behavioral Risk Factor Surveillance System. J Health Econ.

[41] Kato K, Silva MJ, Needham LL, Calafat AM (2005). Determination of 16 phthalate metabolites in urine using automated sample preparation and on-line preconcentration/high-performance liquid chromatography/tandem mass spectrometry. Anal Chem.

[42] Gavin QW, Ramage RT, Waldman JM, She J (2014). Development of HPLC-MS/MS method for the simultaneous determination of environmental phenols in human urine. Int J Environ Anal Chem.

[43] Hornung RW, Reed LD (1990). Estimation of average concentration in the presence of nondetectable values. Appl Occup Environ Hyg.

[44] Mahalingaiah S, Meeker JD, Pearson KR, Calafat AM, Ye X, Petrozza J (2008). Temporal variability and predictors of urinary bisphenol A concentrations in men and women. Environ Health Perspect.

[45] NHANES. 2013-2014: phthalates and plasticizers metabolites - urine data documentation, codebook, and frequencies. 2020. https://wwwn.cdc.gov/Nchs/Nhanes/2013-2014/PHTHTE_H.htm.

[46] NHANES. 2013-2014: personal care and consumer product chemicals and metabolites data documentation, codebook, and frequencies. 2020. https://wwwn.cdc.gov/Nchs/Nhanes/2013-2014/EPHPP_H.htm.

[47] Meeker JD, Calafat AM, Hauser R (2012). Urinary phthalate metabolites and their biotransformation products: predictors and temporal variability among men and women. J Expo Sci Environ Epidemiol.

[48] Shoaff JR, Coull B, Weuve J, Bellinger DC, Calafat AM, Schantz SL (2020). Association of exposure to endocrine-disrupting chemicals during adolescence with attention-deficit/hyperactivity disorder–related behaviors. JAMA Netw Open.

[49] Tewar S, Auinger P, Braun JM, Lanphear B, Yolton K, Epstein JN (2016). Association of bisphenol A exposure and attention-deficit/hyperactivity disorder in a national sample of U.S. children. Environ Res.

[50] Wang H, Zhou Y, Tang C, He Y, Wu J, Chen Y (2013). Urinary phthalate metabolites are associated with body mass index and waist circumference in chinese school children. PLoS ONE.

[51] Trasande L, Attina TM, Blustein J (2012). Association between urinary bisphenol A concentration and obesity prevalence in children and adolescents. JAMA.

[52] Franken C, Lambrechts N, Govarts E, Koppen G, Den Hond E, Ooms D (2017). Phthalate-induced oxidative stress and association with asthma-related airway inflammation in adolescents. Int J Hyg Environ Health.

[53] Just AC, Miller RL, Perzanowski MS, Rundle AG, Chen Q, Jung KH (2015). Vinyl flooring in the home is associated with children’s airborne butylbenzyl phthalate and urinary metabolite concentrations. J Expo Sci Environ Epidemiol.

[54] Watkins DJ, Eliot M, Sathyanarayana S, Calafat AM, Yolton K, Lanphear BP (2014). Variability and predictors of urinary concentrations of phthalate metabolites during early childhood. Environ Sci Technol.

[55] Colacino JA, Harris TR, Schecter A (2010). Dietary intake is associated with phthalate body burden in a nationally representative sample. Environ Health Perspect.

[56] Trasande L, Sathyanarayana S, Jo Messito MS, Gross R, Attina TM, Mendelsohn AL (2013). Phthalates and the diets of US children and adolescents. Environ Res.

[57] Rudel RA, Gray JM, Engel CL, Rawsthorne TW, Dodson RE, Ackerman JM (2011). Food packaging and bisphenol A and bis(2-ethyhexyl) phthalate exposure: findings from a dietary intervention. Environ Health Perspect.

[58] Schecter A, Lorber M, Guo Y, Wu Q, Yun SH, Kannan K (2013). Phthalate concentrations and dietary exposure from food purchased in New York state. Environ Health Perspect.

[59] Fierens T, Van Holderbeke M, Willems H, De Henauw S, Sioen I (2013). Transfer of eight phthalates through the milk chain — a case study. Environ Int.

[60] Geens T, Aerts D, Berthot C, Bourguignon J-P, Goeyens L, Lecomte P (2012). A review of dietary and non-dietary exposure to bisphenol-A. Food Chem Toxicol.

[61] Mendum T, Stoler E, VanBenschoten H, Warner JC (2011). Concentration of bisphenol A in thermal paper. Green Chem Lett Rev.

[62] Lewis RC, Meeker JD, Peterson KE, Lee JM, Pace GG, Cantoral A, et al. Predictors of urinary bisphenol A and phthalate metabolite concentrations in Mexican children. Chemosphere. 2013;93. https://www.ncbi.nlm.nih.gov/pmc/articles/PMC3818401/. 10.1016/j.chemosphere.2013.08.038 PMC3818401 24041567

[63] Braun JM, Kalkbrenner AE, Calafat AM, Bernert JT, Ye X, Silva MJ, et al. Variability and predictors of urinary bisphenol A concentrations during pregnancy. Environ Health Perspect. 2011;119:131–7. 10.1289/ehp.1002366 PMC3018492 21205581

[64] Bemrah N, Jean J, Rivière G, Sanaa M, Leconte S, Bachelot M (2014). Assessment of dietary exposure to bisphenol A in the French population with a special focus on risk characterisation for pregnant French women. Food Chem Toxicol.

[65] Lehmler H-J, Liu B, Gadogbe M, Bao W (2018). Exposure to bisphenol A, bisphenol F, and bisphenol S in U.S. adults and children: The National Health and Nutrition Examination Survey 2013-2014. ACS Omega.

[66] Hehn RS (2016). NHANES data support link between handling of thermal paper receipts and increased urinary bisphenol A excretion. Environ Sci Technol.

[67] Terry MB, Michels KB, Brody JG, Byrne C, Chen S, Jerry DJ, et al. Environmental exposures during windows of susceptibility for breast cancer: a framework for prevention research. Breast Cancer Res. 2019;21:96. 10.1186/s13058-019-1168-2 PMC6701090 31429809

