# Supplementary material for: Dietary intake and household exposures as predictors of urinary concentrations of high molecular weight phthalates and bisphenol A in a cohort of adolescents
Source: J Expo Sci Environ Epidemiol. 2021 Feb 22;32(1):37–47. doi: 10.1038/s41370-021-00305-9 (PMC8380263; doi:10.1038/s41370-021-00305-9)
Supplement: Supplementary file 1 — Supplemental Table 1 [file 41370_2021_305_MOESM1_ESM.docx]

**Supplemental Table 1.** Geometric means (GM) of specific gravity-corrected urinary analyte concentrations (ng/mL) by (yes/no) exposure of predictors with p-value <0.10 in GEE models.

|  | Metabolites | | | | |
| --- | --- | --- | --- | --- | --- |
| Predictor | MBzP | ∑DEHP | MCPP | BPA | |
| **Family’s Usual Practices** | | | | | |
| Plastic containers for leftovers |  |  |  |  | |
| No (12) | 3.9 | -- | -- | -- | |
| Yes (188) | 6.8 | -- | -- | -- | |
| Linoleum or vinyl floors in home |  |  |  |  | |
| No (100) | 5.0 | -- | -- | -- | |
| Yes (100) | 8.7 | -- | -- | -- | |
| Operate cash register at work |  |  |  |  | |
| No | -- | -- | -- | 1.1 | |
| Yes | -- | -- | -- | 1.7 | |
| **Participant’s Recent Practices (past 1-12 hours)** | | | | | |
| **Foods Today** |  |  |  |  | |
| Chicken |  |  |  |  | |
| No (185) | -- | -- | 1.8 | -- | |
| Yes (15) | -- | -- | 3.1 | -- | |
| Cold cuts or deli meats |  |  |  |  | |
| No (191) | -- | -- | 1.8 | -- | |
| Yes (9) | -- | -- | 3.9 | -- | |
| **Drinks Today** |  |  |  |  | |
| Coffee or hot tea |  |  |  |  | |
| No (183) | -- | -- | 1.8 | -- | |
| Yes (17) | -- | -- | 2.4 | -- | |
| **Participant’s Recent Practices (past 24 hours)** | | | | | |
| **Meal Location** |  |  |  |  | |
| Fast food today or yesterday |  |  |  |  | |
| No (114) | -- | -- | 1.7 | -- | |
| Yes (86) | -- | -- | 2.2 | -- | |
